# Supplementary material for: Evaluating the use of the QUiPP app and its impact on the management of threatened preterm labour: A cluster randomised trial
Source: PLoS Med. 2021 Jul 6;18(7):e1003689. doi: 10.1371/journal.pmed.1003689 (PMC8291648; doi:10.1371/journal.pmed.1003689)
Supplement: S1 File — sPTB, spontaneous preterm birth; TPTL, threatened preterm labour. (DOCX) [file pmed.1003689.s001.docx]

Supplementary File 1:

Table of clinical details of spontaneous preterm births within 7 days of TPTL presentation which were not appropriately managed during EQUIPTT trial

| Case | Site | QUiPP risk | Delivery gestation (weeks^days^) | Clinical Situation/impact | Neonatal |
| --- | --- | --- | --- | --- | --- |
| 1 | Intervention | 0.2% | 30^+2^ | No steroids or admission- but re-attended and received timely ACS | NNU admission |
| 2 | Intervention | 0.5% | 33^+0^ | Not admitted but re-attended and received timely ACS | NNU admission |
| 3 | Intervention | 0.6% | 33^+3^ | Not admitted but re-attended and received timely ACS | NNU admission |
| 4 | Intervention | 16.4% | 31^+4^ | Admitted but ACS previously (not within 7 days) | NNU admission |
| 5 | Control | | 34^+4^ | Not admitted, ACS previously (not within 7 days), | NNU + sepsis |
| 6 | Control | | 25^+2^ | No ACS, twins- | Ex-utero transfer |
| 7 | Control | | 29^+3^ | No ACS, reattended and received magnesium sulphate | Neonatal death |
| 8 | Control | | 32^+3^ | No ACS or admission | Nil |
| 9 | Control | | 33^+0^ | No ACS, Delivered same day | NNU admission |
| 10 | Control | | 35^+3^ | No admission or ACS | Nil |
| 11 | Control | | 34^+6^ | No admission but received ACS | NNU admission |
| 12 | Control | | 34^+2^ | No steroids, Delivered same day | NNU admission |
| 13 | Control | | 33^+1^ | Twins, Admitted 2/3 episodes, ACS not within 7 days | NNU- both twins RDS |
| 14 | Control | | 33^+5^ | Admitted but no ACS | NNU admission, left hypoplastic heart syndrome |
| 15 | Control | | 28^+1^ | Admitted but ACS previously (not within 7 days) | NNU admission, RDS |
| 16 | Control | | 27^+4^ | Admitted but ACS previously (not within 7 days) | NNU admission, RDS |
