# Supplementary material for: Evaluating the use of the QUiPP app and its impact on the management of threatened preterm labour: A cluster randomised trial
Source: PLoS Med. 2021 Jul 6;18(7):e1003689. doi: 10.1371/journal.pmed.1003689 (PMC8291648; doi:10.1371/journal.pmed.1003689)
Supplement: S1 Fig — QUIPP, QUantitative Innovation in Predicting Preterm birth. (PPTX) [file pmed.1003689.s008.pptx]

## Slide 1
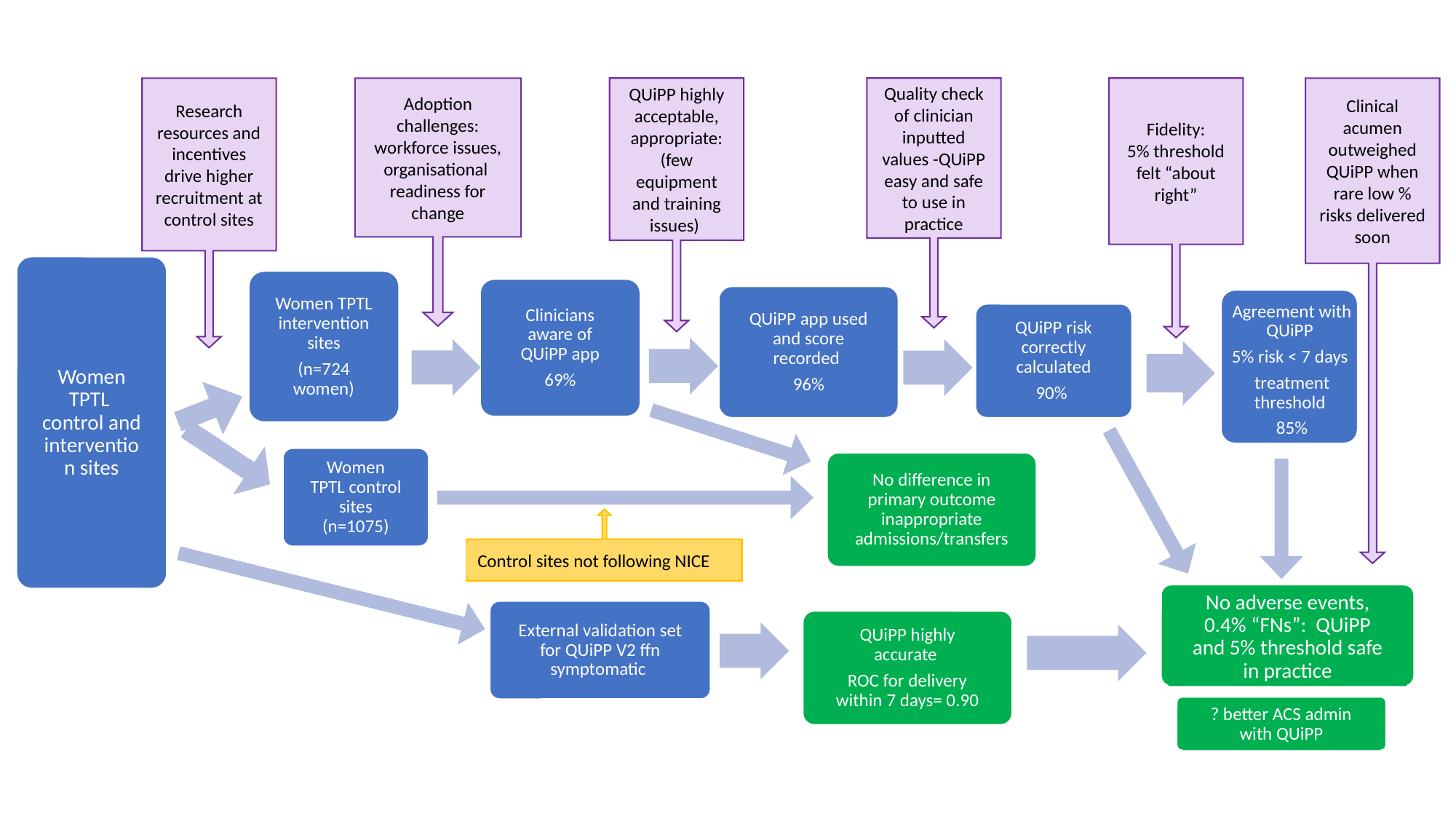

Research resources and incentives drive higher recruitment at control sites
Adoption challenges: workforce issues, organisational readiness for change
QUiPP highly acceptable, appropriate: (few equipment and training issues)
Quality check of clinician inputted values -QUiPP easy and safe to use in practice
Fidelity:
5% threshold felt “about right”
Clinical acumen outweighed QUiPP when rare low % risks delivered soon
Women TPTL control and intervention sites
Women TPTL intervention sites
(n=724 women)
Clinicians aware of QUiPP app
69%
QUiPP app used and score recorded
96%
Agreement with QUiPP
5% risk < 7 days
treatment threshold
85%
QUiPP risk correctly calculated
90%
Women TPTL control sites (n=1075)
No difference in primary outcome inappropriate admissions/transfers
Control sites not following NICE
No adverse events, 0.4% “FNs”: QUiPP and 5% threshold safe in practice
External validation set for QUiPP V2 ffn symptomatic
QUiPP highly accurate
ROC for delivery within 7 days= 0.90
? better ACS admin with QUiPP
