# Supplementary material for: Evaluating the use of the QUiPP app and its impact on the management of threatened preterm labour: A cluster randomised trial
Source: PLoS Med. 2021 Jul 6;18(7):e1003689. doi: 10.1371/journal.pmed.1003689 (PMC8291648; doi:10.1371/journal.pmed.1003689)
Supplement: Attachment — Submitted filename: PLosMedEQUIPTTRevComms.9_2_21 .docx [file pmed.1003689.s009.docx]

We thank the editors and reviewers for their constructive comments, which have all been addressed individually below.

Requests from the editors:

*As noted by one reviewer - can the ISRCSN registry details for the trial (id - ISRCTN17846337; link - <http://www.isrctn.com/ISRCTN17846337>) be included within the submitted paper (ideally at end of abstract) - this is our usual style.

P3, 12 - Done

*The issue of individual informed consent (lack of) for this trial came up, and we recognise that the authors currently say in the paper that individual consent would not be feasible given the way the trial was conducted, and it's recognised for some cluster RCTs that individual consent can be waived. But it would be good as well to clarify (and state in the paper) whether the ethics committee specifically waived the need for informed consent.

P6 l22 Added: “The no-consent model was specifically addressed in the ethics application and the ethics committee waived the need for informed consent (REC reference 17/LO/1802)”

*We'd suggest revising the title per the journal style, where these normally have a two-part format separated by colon (question/objective: study design) - example <https://journals.plos.org/plosmedicine/article?id=10.1371/journal.pmed.1002949> (Implementing a structured model for osteoarthritis care in primary healthcare: A stepped-wedge cluster-randomised trial)

P1L1 Changed to “Evaluating the use of the QUiPP app and its impact on the management of threatened preterm labour: a cluster-randomised trial.”

*At this stage please now restructure the abstract into the journal style - the subheadings for research papers should be Background, Methods and Findings, Conclusions (Methods and Findings is a single subhead). Within each subsection please use full sentences rather than (partial) sentence fragments.

Done

*In the last sentence of the Abstract Methods and Findings section, please include a note about any key limitation(s) of the study's methodology.

Added “An insufficient number of clusters who followed national guidance at the control sites, and limitations in implementation of the QUiPP app contributed to the intervention’s null effect on the primary outcome.”

*We noticed a few typos/manuscript preparation errors, so please check through carefully before resubmitting the revision. Example in abstract - "Intervention sites were assigned to use the QUiPP app (with quantitative fetal fibronectin and/or or cervical length with history)in and suggested admission..." (the "in and suggested admission" part doesn't quite make sense.

Done. Also changed “inappropriate” to “unnecessary” management due to recent feedback that “inappropriate” sounds judgemental.

*Please refer to the CONSORT for cluster trials reporting guideline (<https://www.equator-network.org/reporting-guidelines/consort-cluster/>) and it might be helpful to add more detail on some aspects of reporting both to the paper (see reviewer 2's comments), and to the abstract, for example what the unit of randomization was (mat unit?) and how many were assigned to each arm. For submitted trials the journal normally wants to see a completed CONSORT checklist appended as supporting information - so we'd recommend adding this (and the CONSORT for cluster RCTs would probably be the most helpful here).

Done and added as supplementary file

*Some minor changes to the writeup would also be helpful, we'd suggest moving the "Aims and Objectives" subsection into Methods and also moving the Ethics Approval information from the end of the paper to the end of the Methods.

Done

*At this stage, we ask that you include a short, non-technical Author Summary of your research to make findings accessible to a wide audience that includes both scientists and non-scientists. The Author Summary should immediately follow the Abstract in your revised manuscript. This text is subject to editorial change and should be distinct from the scientific abstract. Please see our author guidelines for more

Done – added

Accurate diagnosis of premature labour is desirable in order ensure optimum management of those most at risk of preterm birth and to prevent the maternal and fetal risks incurred by unnecessary interventions to the majority of women who do not deliver within a week of presentation. The QUIPP App is a clinical decision-making aid based on woman’s individual risk factors for premature birth and quantitative fetal fibronectin (fFN) values and/or cervical length as determined by transvaginal ultrasound, both of which are existing point-of-care tests. This trial randomised maternity units to use the QUiPP app to triage women with symptoms of premature labour versus the unit’s conventional management. The study also provided a dataset to test the accuracy of the QUiPP app on a population other than that on which it was built. The trial provided evidence that the QUiPP is accurate and safe: no women missed timely treatments with its use. However, the QUiPP intervention did not succeed in lowering unnecessary admission and transfer decisions relative to the control sites. The authors believe this was due to larger than anticipated non-compliance with national guidance, which does not recommend predictive tests below 30 weeks’ gestation, and problems in ensuring all clinicians used the app at interventions sites.

-----------------------------------------------------------

Comments from the reviewers:

Reviewer #1: It is a relevant study as presently, AI based algorithms are being used increasingly in medical practice to improve decision making.

Thank you for your supportive feedback

-----------------------------------------------------------

Reviewer #2: Statistical review

This paper reports a cluster randomised trial comparing use of an app to guide decision about whether to admit pregnant women at risk of pre-term birth. There were no significant differences between the arms, which appears to be due to control sites using qfFN, which is a major contributor to the app risk score.

I did not see a protocol provided, or details of whether the trial was registered on a repository such as [clinicaltrials.gov](http://clinicaltrials.gov), which is required for PLOS medicine.

P7l3- the protocol was published in BMC Pregnancy and Childbirth in 2019 and we reference it here. We have added the trial registry number following the abstract as per Editor’s comments

My other comments were more minor:

1.	Abstract - I found it hard to follow line 15 and 16 of the abstract, in particular the part outside the brackets.

We agree with the reviewer and have altered the abstract format for Editor’s comments. Have removed brackets as the constituents of QUiPP are now outlined in Background, Hopefully the following simpler to read - *“Pregnant women (n=2430) between 23^+0^ and 34^+6^ weeks’ gestation with symptoms of preterm labour were assigned to either the intervention or control. The intervention was the use of the QUiPP app with admission, antenatal corticosteroids and transfer advised for women with a QUiPP risk of delivery > 5% within 7 days.”*

2.	Abstract, main outcome measures - I think it's a bit unclear here whether the outcomes described here are separate primary outcomes or combined into one. Later in the paper it's clear it's the latter but here I was confused by 'main outcome measures' as the sub-title.

Agreed- the confusing subtitle has gone and have emphasised composite primary outcome…” Unnecessary management for TPTL **was a composite primary outcome** defined by the number of unnecessary admission decisions (admitted and delivery interval >7 days OR not admitted and delivery interval ≤7 days) and the **sum** of unnecessary in-utero transfer decisions/actions (in-utero transfer that occurred or were attempted >7 days prior to delivery, and ex-utero transfers that should have been in-utero (attempted and not attempted). “

3.	Abstract, main outcome measures - it's also not too clear whether the outcome was binary, i.e. occurrence of one or both of the events, or as written, the sum of number of events. I would recommend this is made clearer. A similar comment applies to the methods section on page 7.

Corrected – within response to q2

4.	Abstract - for the primary outcome I would recommend a p-value is included as well as the confidence interval.

Added confidence intervals

5.	Page 5 - can more information be provided about the randomisation procedure and how it was performed?

P5 l31 -

Randomisation was performed at the cluster (maternity centre) level. A computer-generated random allocation sequence was used; stratified by the level of special care available. Randomisation was designed to allocate approximately equal numbers to each arm according to the level of care provided: neonatal intensive care unit vs local neonatal unit or special care baby unit. Due to their being an odd number of centres, 7 sites were allocated to one group and 6 to the other.

6.	Page 8 - the statistical analysis says that risk ratios were calculated but the abstract reports odds ratios. This section also mentions 'minimisation process' which should be described more in the randomisation section as per previous comment.

Despite what the protocol says, we opted for a multi-level model. In Stata, this implies the command -melogit-, which gives odds ratios.  Using binomial regression with a log link to give risk ratios, with a correction for level of care, RR and OR were very similar - RR 1.00 (0.56 to 1.76) vs OR 0.97 (0.66 – 1.42)

P8l11- changes to *Whilst our protocol planned to express treatment effects as risk ratios (relative risk), at analysis we opted for a multi-level model in Stata which provides odds ratios with 95% Confidence Intervals (CI), using binomial regression and adjusting for variables used in the minimization process.* .

7.	Page 8 - I would recommend providing more details of the statistical analysis for evaluating the predictive accuracy of the QUiPP app.

Thank you for pointing out this omission. We have added

P9l9

*We have previously described the creation of the prognostic models for the QUiPP app v.2 symptomatic algorithms, the simple calibration of these models and a temporal internal validation. Using % risk of ≥ 5% as an indication of a positive test, the Area under the Curve for prediction of preterm birth within 7 days using the fFN algorithm was 0.893 (4). However external validation is required to test the generalisability of the prognostic models and correct for over-fitting. To calculate the performance of the QUiPP app symptomatic qfFN predictive model, a validation set was created from EQUIPTT participants with a qfFN value from the duration of the trial period across all 13 sites. Any qfFN values (n=1) documented as >500 were changed to 500 (as the qfFN machine will not provide a value >500 ng/mL).*

8.	Figure 1 - it would be of interest to know whether the loss-to-follow-up was similar between the two groups. Maybe this could be given by arm in the figure?

Those lost to follow-up were excluded prior to analysis and the 104 include women in the run-in and post-analysis period so this edit is likely to confuse Figure 1. However, we agree this is relevant given our findings and have added the following to the text

*p12/5 The lost to follow-up rate was 5% (37/761) in intervention sites and 3% (36/1111) in control sites.*

9.	Page 11 - the authors alternate between using 'cluster' period and 'analysis' period which I found a bit confusing - are these the same things?

Apologies- corrected to “cluster analysis period” for clarification

10.	Table 3 - were the neonatal outcomes not planned to be analysed statistically? If so then I'd provide the results as in table 2.

We did not power for neonatal outcomes, so did not test for statistical significance. Clinically it is useful to view these outcomes independently from the baseline characteristics table.

11.	Page 13 - I found the per-protocol analysis difficult to follow: could this be described more in the methods section?

Apologies- have added the below to Methods to explain rationale

P8l29 We anticipated that the cluster design (institutional level) approach to randomisation of the QUiPP intervention would require a high uptake and adoption of the intervention; this was not a problem for control sites as they were continuing usual care. To estimate the contribution of this effect, we also planned a per protocol analysis to evaluate how the primary outcome would be impacted by ideal use of the QUiPP app, i.e. on all eligible episodes of threatened preterm labour and with adherence to the 5% threshold to guide management decisions.

Also simplified Results introduction of this analysis

*P14/3 “As per our pre-specified per-protocol analysis, the proportion of unnecessary admissions and discharges were calculated for women as if the 5% threshold was always followed with the given QUiPP risks.”*

James Wason

-----------------------------------------------------------

Reviewer #3: 

This cluster trial offers a deep insight on the clinical management of threatened PTL, in UK.
The planning and execution of the study have been well performed.

Overall, a 17% PTB rate in women presenting (admitted or not) for PTL are within expected range in settings of good quality of care, not being affected by excessive fear and anxiety.

The trial demonstrated that clinicians behave better than guidelines provided for them! Certainly, app would warrant a more standardized application of risk factors.

I congratulate Authors for this very interesting study.

Thank you for your positive comments

Few points to better understand data:

1)	There should be an explanation why lot of tertiary centres pertain to control. It was expected that standard of cares could be better than in other units. Thus we believe app will be less added value?

Yes, this was our interpretation. We alluded to this on P2/25 “The randomisation of the most research-active tertiary centres to the control arm is likely to have distorted this degree of necessary management, as many were using qFFN and components of the app already; we could not mitigate against the app being used informally in the control arm.” But have now added for clarification `’It may be that QUiPP has less added value in sites which already use qfFN judiciously, relative to those using the qualitative 50 ng/mL cut-off to direct management or those with a treat-all strategy ”

2)	How many centres used  "old" yes/not  fFN test? In the case, is possible a comparison between quali- and quantitative test on main outcome?

The decision for the control arm to reflect local conventional management of threatened preterm labour, aimed to explore actual practice since the NICE 2015 guidance to treat-all <30 weeks’ gestation. Whilst qualitative fFN was the most popular test at control arms, one site used Partosure. Also, the local protocols were varied regarding when fFN was indicated and its correlation with cervical assessment or ultrasound findings. In addition, whilst largely driven by quantitative fFN, the QUiPP risk will vary with risk factors and gestation- so the intervention arm cannot be equated with quantitative fFN alone. These issues restrict a direct comparison of qualitative fFN and quantitative fFN, but given that qualitative fFN was the most common test used at control sites - the low unnecessary management rates in both arms supports both qualitative fFN and QUiPP as discussed in p21,4.

3)	How was steroids rate administered after 34 weeks?

Whilst this is an interesting area. this was not analysed for this study as fFN is not used after 35 weeks and different priorities are likely to drive decisions to give steroids in late preterm gestations.

P13l2 for clarity regarding definition of unnecessary steroids, we have added “*ACS were defined as appropriate if at least one dose was administered ≥24 h and ≤7 days before a delivery which occurred <36 weeks. ACS was classified as unnecessary if there was >7 days between time first dose was administered and delivery.”*
-----------------------------------------------------------

Reviewer #4: 

The objective of this multi-centre cluster randomised-controlled trial was to investigate the impact of the use of the QUiPP app versus conventional management on inappropriate management for threatened preterm labour (TPTL). According to the intention to treat analysis, inappropriate management of TPTL was 11.3% at the intervention sites versus 11.5% at control sites (OR 0.97; 95% CI 0.66-1.42). According to the per protocol analysis, inappropriate admissions and discharges were also not significantly reduced in the intervention arm (7.4% versus 9.9%).

This is an interesting randomised-controlled trial which should be considered for publication. However, I have several comments.

The methodology of this RCT is adequate with a low risk of bias:

Internal validity:

- A selection bias is very unlikely: EQUIPTT was a cluster randomised-controlled trial with a parallel group design across 13 obstetric centres: randomisation was performed at the cluster (maternity centre) level.
- Because masking was not feasible, performance bias is possible but unlikely because the QUiPP app was only implemented in the intervention centres and management of women was similar between the two arms.
- A detection bias is very unlikely because the composite primary outcome of inappropriate management for TPTL was objective and defined by the number of inappropriate admission decisions: admitted and delivery interval >7 days or not admitted and delivery interval ≤ 7 days, and the number of inappropriate in-utero transfer decisions/actions: IUTs that occurred or were attempted >7 days prior to delivery, and ex utero transfers that should have been in-utero.
- An attrition bias is possible but also unlikely: indeed, 1872 women (761 intervention and 1111 control) were recruited but only 1799 women (724 intervention and 1075 control) were included in the ITT analysis.

External validity : 

13 obstetric centres including Special Care Unit (level 1), local Neonatal Unit (level 2) and Neonatal Intensive Care Unit (level 3) participated in this study, ensuring an excellent external validity.

Thank your positive assessments of bias and validity

However, this paper presents several limitations that should be considered:

The per protocol analysis is interesting to report. However, this analysis is not specified in the Methods section (Statistical Analysis). The authors should clarify whether this is a prespecified analysis or a post-hoc analysis.

This was a pre-specified analysis and the following has been added to the statistical analysis plan

*P8l29 “We anticipated that the cluster design’s randomisation of the QUiPP intervention at the institutional level relies on high uptake and adoption of the intervention, which is not a problem for control sites continuing usual care. To estimate the contribution of this effect, we also planned a per protocol analysis to evaluate how the primary outcome would be impacted by ideal use of the QUiPP app, i.e. on all eligible episodes of threatened preterm labour and with adherence to the 5% threshold to guide management decisions.”*

Also simplified Results introduction of this analysis

*P14/3 “As per our pre-specified per-protocol analysis, the proportion of unnecessary admissions and discharges were calculated for women as if the 5% threshold was always followed with the given QUiPP risks.”*

A main strong of this RCT is its pragmatic approach and the absence of significant statistical differences between the intervention and control arms, both in ITT analysis and per protocol analysis reinforces the reliability of the results. Therefore, the post-hoc analysis evaluating the impact of the QUiPP app compared with current UK national guidance is not consistent with the pragmatic approach of the authors and should be deleted. This post-hoc analysis could even be considered a "spin" leading the authors to consider the usefulness of the QUiPP app while their trial is negative and shows the lack of benefit to the use of the QUiPP app in real life.
Therefore, The Results and the Discussion Sections should be reviewed, mainly focusing on the prespecified analysis reporting negative results. I understand that the authors are disappointed by this negative result but this "spin" has the effect of devaluing the methodological quality of this trial.

We thank the reviewer for this frank appraisal, and are happy to lose this post-hoc analysis from the paper given his concerns- removed from Results and Discussion.

Conclusion changed to

*This cluster randomised trial did not demonstrate that use of the QUiPP app reduced unnecessary management of threatened preterm labour compared to current management. However, the management of TPTL in the control arms was not according to national guidance and may not have reflected wider practice. The low rates of overtreatment or missed events in either arms, support interpretation of qfFN, with or without the QUiPP app, as a safe and accurate method for identifying women most likely to benefit from PTL interventions. It also highlights the need for greater consideration of implementation strategies for this type of research study.*

Patrick Rozenberg
